# Response to Mughal et al, re: “The Biomedical Publications Industry Must Change to Better Serve the Needs of Science and Scientists”

**DOI:** 10.20411/pai.v10i2.835

**Published:** 2025-06-12

**Authors:** Michael M. Lederman, Neil S. Greenspan

**Affiliations:** 1 Case Western Reserve University School of Medicine, Cleveland, Ohio

We thank Abeera Saleem Mughal and Hafiz Shahbaz Zahoor for their letter regarding our recent editorial “The Biomedical Publications Industry Must Change to Better Serve the Needs of Science and Scientists” [1]. Drs. Mughal and Zahoor are concerned that researchers from low- and middle-income countries face challenges to publication; we agree. By eliminating fees for access and fees for publication, *Pathogens and Immunity* has removed some of these barriers. They propose that Open Peer Review also could assure greater fairness in reviews, but to our mind, the risk of insufficient candor with this approach to peer review was not one the editors were willing to take. Drs. Mughal and Zahoor asked for a practical roadmap for implementing our journal policies. The roadmap of our policies and procedures is available right on our journal website. Also, the editors of *Pathogens and Immunity* are prepared to offer (and have provided) advice and assistance to other journals interested in adopting our approach.

Drs. Mughal and Zahoor conclude by noting that our journal cannot change the world and that a system-wide change in biomedical publications policies is necessary. We acknowledge limitations to what one group of editors can accomplish, but we are not aware of a global authority that can yet bring about the desired transformation in biomedical publishing. At present, we have offered our journal and its policies as an example to other journals and journal editors who would like to make life better for scientists and authors.
